# Plasmonic waveguide design for the enhanced forward stimulated brillouin scattering in diamond

**DOI:** 10.1038/s41598-017-18507-3

**Published:** 2018-01-08

**Authors:** Qiang Liu, Luigi Bibbó, Sacharia Albin, Qiong Wang, Mi Lin, Huihui Lu, Zhengbiao Ouyang

**Affiliations:** 1 THz Technical Research Center of Shenzhen University, Shenzhen, 518060 China; 2 College of Electronic Science & Technology, Shenzhen University, Shenzhen, 518060 China; 3 Engineering Department, Norfolk State University, Norfolk, Virginia 23504 USA; 4 Department of Optoelectronic Engineering, Jinan University, Guangzhou, 510632 China

## Abstract

We propose a scheme of metal/dielectric/metal waveguide for the enhanced forward stimulated Brillouin scattering (FSBS) in diamond that is mediated by gap surface plasmons. Numerical results based on finite-element method show that the maximum Brillouin gain in the small gap (~100 nm) can exceed 10^6^ W^−1^ m^−1^, which is three orders of magnitude higher than that in diamond-only waveguides. It is found that the radiation pressure that exists at the boundaries of metal and diamond plays a dominant role in contributing to the enhanced forward stimulated Brillouin gain, although electrostrictive forces interfere destructively. Detailed study shows that high FSBS gain can still be obtained regardless of the photoelastic property of the dielectric material in the proposed plasmonic waveguide. The strong photon-phonon coupling in this gap-surface-plasmon waveguide may make our design useful in the development of phonon laser, RF wave generation and optomechanical information processing in quantum system.

## Introduction

Forward stimulated Brillouin scattering (FSBS)^1–8^ is one kind of light-matter interaction in which the pump photons, Stokes photons and phonons have strong optomechanical coupling that is mediated by optical forces (including radiation pressure (RP) and electrostrictive (ES) forces). Unlike backward stimulated Brillouin scattering (BSBS) where longitudinal phonons participate, FSBS involves transverse phonons.

FSBS was studied first in conventional optical fibers^1^; it was found that Stokes signals in the system were usually too weak due to the very limited confinement of phonons (the wavevector *k* ≈ 0) that were mediating the FSBS process. In order to realize a strong FSBS, extensive studies have been conducted on photonic crystal fibers (PCF)^2,3^, hybrid photonic-phononic waveguides^4–6^ and suspended dielectric photonic wires^7–10^. For example, air-silica microstructure in PCF can selectively localize several transverse guided acoustic modes with operational frequency up to 2 GHz^2^. However, the tunability of optical and acoustic dispersion in the PCF is limited; therefore hybrid photonic-phononic waveguides^4–6^ have been proposed. In suspended nano-scale silicon wires^7,8^, large electric fields existing at the nano-scale boundary of dielectric material and air can boost both the RP and ES forces, which produce tremendous enhancements to the FSBS. It is noted that aforementioned optical fibers or waveguides confine light within diffraction limit, as dielectrics have been used as the medium. If the gain in such systems relies mainly on the ES force, which is the result of the intrinsic photo-elastic property of the nonlinear material, further promotion of FSBS amplification may become quite limited.

In this work, we introduce a gap-surface-plasmonic (GSP) waveguide, in which subwavelength localization is achieved in very narrow gap width^11,12^, in diamond: (1) to produce large RP forces that acts at the dielectric-gap boundaries, which contribute to the main gain to FSBS, (2) to explore a new avenue of tailoring mechanical modes, and (3) to optimize the FSBS gain. The following simulations on full-vector analysis between photonic and elastic modes are performed by using finite-element method (FEM) (COMSOL Multiphysics).

## Structure

**Figure 1:**
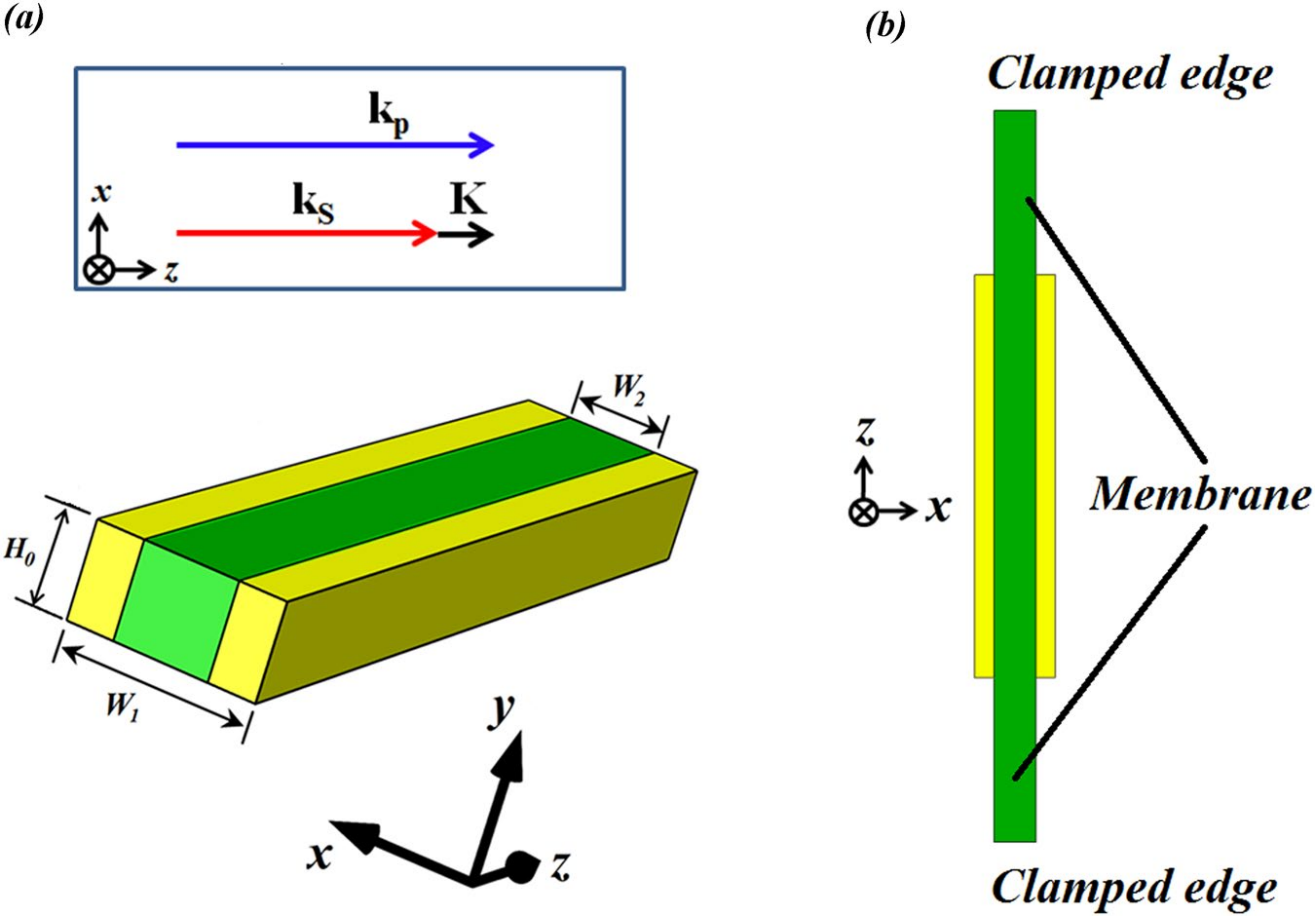
(**a**) Schematic of GSP waveguide; here the yellow and green colors respectively denote silver and diamond, and the background is air; (**b**) Schematic of the suspended waveguide, where the membrane is considered to be formed by increasing the length (along *z* direction) and thickness (along *y* direction) of dielectric material, and the suspension could be achieved by clamping both far edges facing toward *z* direction to the ground material. Note that phase matching condition in FSBS should be satisfied: **k**_**p**_ = **k**_**S**_ + **K**. Here **k**_**p**_, **k**_**S**_ and **K** represent the optical pump, Stokes, and phonon wavevectors respectively. As indicated in ref.^9^, the phase matching condition can be automatically satisfied in FSBS.

We investigate a waveguide geometry consisting of a metal/dielectric/metal as shown in Fig. 1. Figure 1(a) shows the modeling structure, while Fig. 1(b) is a practical structure that can be anchored at the two ends of the extended dielectric membrane facing the *z* direction. More metal nano-/micro-layers (yellow region) could be added on the membrane surfaces as shown in Fig. 1(b). If we use a suspended design of Fig. 1(b) in which both far edges in *z* direction can be clamped, it can prevent phononic energy dissipation, which helps to increase the lateral confinement of elastic modes^7,8^.

We choose diamond as the dielectric membrane that is coated with silver layers on its side-walls. As is well-known, diamond is useful for optomechanical applications, due to its good optical, mechanical and thermal properties, such as wide bandgap (5.48 eV)^10^, high Young’s modulus^13^, high thermal conductivity^14^ and low thermal expansion coefficient^15^. Besides, diamond is perfect for hosting color centers, including negative nitrogen vacancy (NV^−^) center^16^ and silicon vacancy color center^17^. Other candidates for dielectric material could be silicon, polymer, LiNbO_3_, and TeO_2_ which have wide application in photoelastic devices, due to the well-established elastic coefficients. An alternative is to use metal/ferromagnetic dielectric bismuth iron garnet (BIG)/metal to study lattice vibrations, which may lead to interesting application in ultrafast acousto-magneto-plasmonics^18^. We use Young’s modulus *E* = 1035 GPa, Poisson’s ratio *ν* = 0.20, density *ρ*_diamond_ = 3520 kg/m^3^, photo-elastic coefficients (*p*_11_ *, p*_12_ *, p*_44_) = (−0.277, 0.058, −0.171) for diamond^13^, while *c*_11_ = 124 GPa, *c*_12_ = 93 GPa, *c*_44_ = 46 GPa, density *ρ*_silver_ = 10490 kg/m^3^ for silver, as it is anisotropic in mechanical property^19^. The refractive index of diamond *n* = 2.417^20^ and the permittivity of silver is −14.683 + *i*·1.2073 at the incident wavelength 637 nm^21^, which corresponds to the zero phonon line emission from NV^−^ centers embedded in diamond. In all simulations, we consider the width of the silver film equal to the height of the waveguide: *w*(Ag) = (*W*_1_ − *W*_2_)/2 = *H*_0_, which means the cross-sectional view of the silver layer is a square.

## Results and Discussions

**Figure 2:**
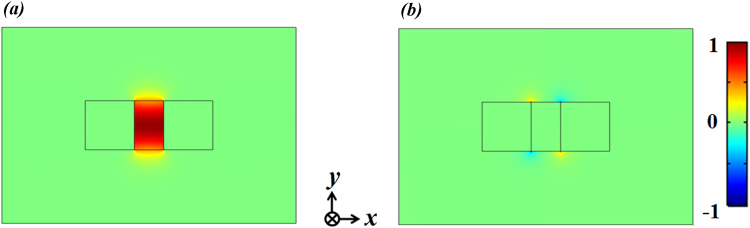
Electric field distributions of (**a**) *E*_*x*_ and (**b**) *E*_*y*_ components from cross-sectional view (*x-y* plane). Geometrical parameters are: *H*_0_ = 250 nm, *W*_2_ = 150 nm. The incident wavelength is 637 nm, which corresponds to the zero phonon line emission produced by nitrogen vacancy that is embedded in diamond.

Both pump and Stokes waves are fundamental transverse-magnetic (TM) waveguide modes. *E*_*x*_ and *E*_*y*_ field distributions of TM modes are shown in Fig. 2(a,b), respectively. Note that for the FSBS, due to the symmetry of the structure (e.g. the waveguide has mirror symmetry about the planes *x* = *W*_1_/2 and *y* = *H*_0_/2), the pump and Stokes waves travel along the same axis while maintaining a co-directional coupling. As indicated in ref.^11^, the structural symmetry affects the modal overlap of optical and elastic vibration modes, resulting in high or low Brillouin gain.

**Figure 3:**
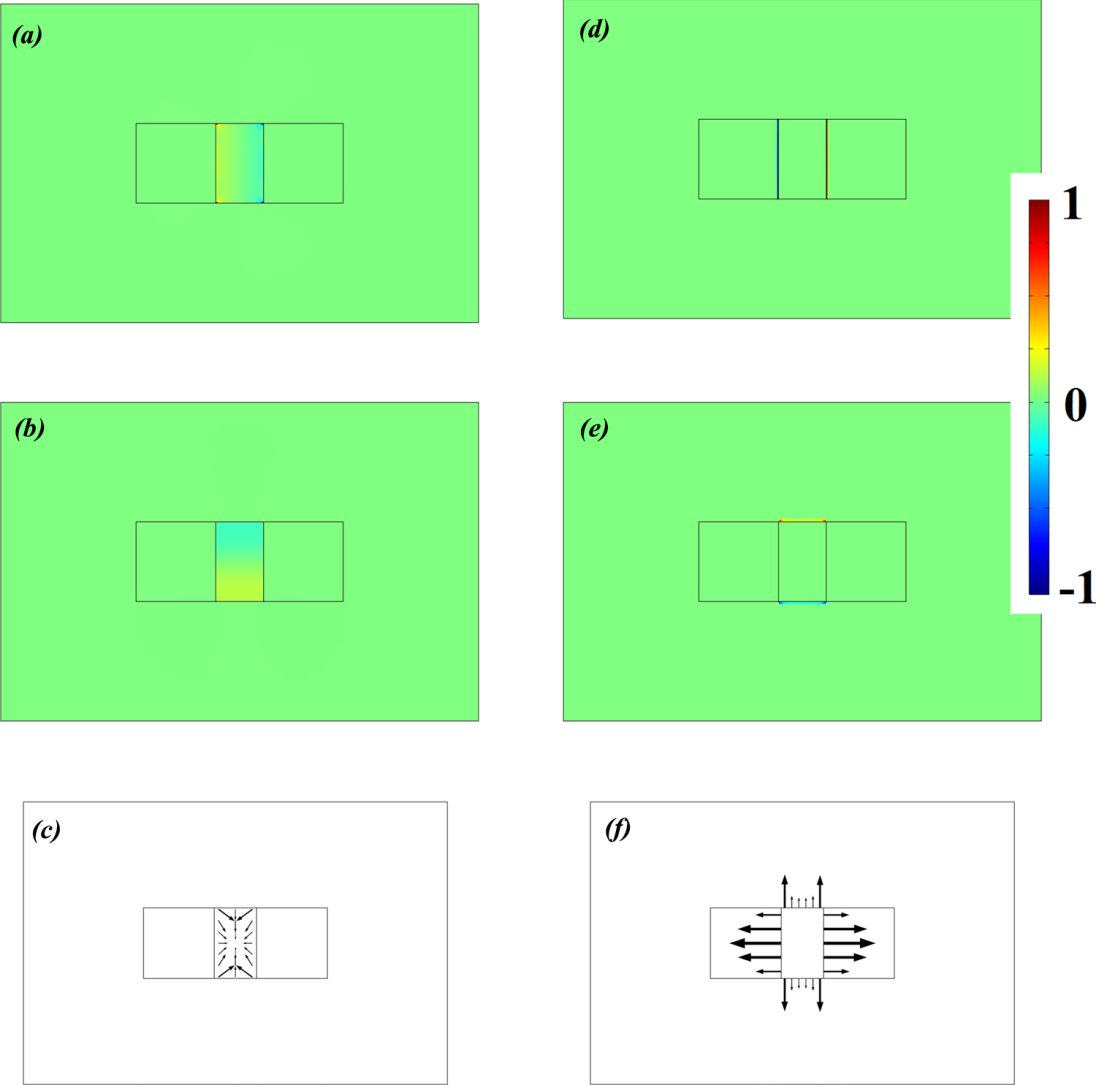
Normalized force distribution of (**a**) ES force in *x* direction and (**b**) in *y* direction. (**c**) Direction of ES forces. (**d**) RP (*x* direction) at vertical boundaries; (**e**) RP (*y* direction) at horizontal boundaries; (**f**) Direction of RP forces. Geometrical parameters are the same as that shown in Fig. 2.

Figures 3(a,b) show the normalized force distribution of ES forces *f* ^*es*^ (that are related to the photo-elastic coefficient of the material) with *x* and *y* components respectively, while Fig. 3(c) shows the direction of *f* ^*es*^. Note that ${f}_{j}^{es}={\partial }_{j}{\sigma }_{ij}$, where *σ*_*ij*_ is the stress tensor induced by electrostriction. In the diamond waveguide, we consider two components: *σ*_*xx*_ and *σ*_*yy*_, where *x*-direction is considered to be the same as [100] crystal direction in diamond. Since diamond shows cubic crystal symmetry, *σ*_*xx*_ and *σ*_*yy*_ can be given as^22^:

1 $${\sigma }_{xx}=-\frac{1}{2}{n}^{4}{\varepsilon }_{0}\cdot [{p}_{11}{|{E}_{x}|}^{2}+{p}_{12}({|{E}_{y}|}^{2}+{|{E}_{z}|}^{2})]$$

2 $${\sigma }_{yy}=-\frac{1}{2}{n}^{4}{\varepsilon }_{0}\cdot [{p}_{11}{|{E}_{y}|}^{2}+{p}_{12}({|{E}_{y}|}^{2}+{|{E}_{z}|}^{2})]$$

Here *n* is the refractive index of diamond, and *ε*_0_ is the permittivity of vacuum.

Figure 3(d,e) show the normalized force distributions of RP forces *f* ^*rp*^ in *x* and *y* directions respectively. RP forces are related to the optical reflection or scattering that happens at the boundary, as derived from Maxwell stress tensor. Figure 3(f) gives the direction of *f* ^*rp*^. Note that ${f}_{j}^{rp}={\partial }_{j}{T}_{ij}$, where the Maxwell stress tensor *T*_*ij*_ is given by:

3 $${T}_{ij}={\varepsilon }_{0}\varepsilon [{E}_{i}{E}_{j}-\frac{1}{2}{\delta }_{ij}{|E|}^{2}]+{\mu }_{0}\mu [{H}_{i}{H}_{j}-\frac{1}{2}{\delta }_{ij}{|H|}^{2}]$$

In Eqn. (3), *E*_*i*(*j*)_ (*H*_*i*(*j*)_) denotes the *i*(*j*)-th component of electric (magnetic) field, *μ*_0_ is the permeability of vacuum, while *ε*(*μ*) denotes the relative permittivity (permeability).

To quantitatively describe the average forces acting on the dielectric boundaries, another parameter termed as spatially averaged radiation pressure (SARP) is introduced, which can be expressed as:

4 $${\overline{f}}^{rp}=({\overline{f}}_{x}^{rp}+{\overline{f}}_{y}^{rp})/C$$

where *C* is the half perimeter of the cross-section of the dielectric material. Here the power normalized radiation pressure forces in *x* and *y* directions are defined as: ${\overline{f}}_{x}^{rp}={\overline{T}}_{xx}\cdot H/P$, and ${\overline{f}}_{y}^{rp}={\overline{T}}_{yy}\cdot {W}_{2}/P$, where *P* is the guided power. ${\overline{T}}_{xx}$ and ${\overline{T}}_{yy}$ are the spatially averaged stress induced by RP, which follows the definition^22^:

5 $${\overline{T}}_{ij}=\frac{1}{{A}_{wg}}{\iint }_{{A}_{wg}}{T}_{ij}\cdot dxdy$$

Here *A*_*wg*_ is the cross-sectional area of the dielectric material.

As seen in Fig. 3(c,f), irrespective of *x* or *y* direction, the RP and ES forces add destructively, which in turn decreases the total forces on both the horizontal (*y*-direction) and vertical boundaries (*x*-direction). However, further calculations show that the *x* component of RP is significantly increased due to: (1) the strong enhancement of electric fields because of the sub-wavelength gap surface plasmon confinement, and (2) the abrupt change of Maxwell tensor *T*_*xx*_ at the boundaries between silver and diamond. Note that silver can be considered as a perfect electric conductor (PEC), and the electric field in it is zero, which means that the *T*_*xx*_ at the vertical boundary (*x*-direction) of silver side vanishes. The ES forces in *x* and *y* directions (see Fig. 3(c)) are shown to have opposite signs compared with RP forces (see Fig. 3(f)), such an interesting phenomenon, though uncommon, is also found in the recently published works on silicon^7–9^. This result is explained as follows: as a large portion of the electric energy is carried by the *E*_*x*_ component in this GSP waveguide, *f* ^*es*^ is strongly dependent on *p*_11_ and *p*_12_ coefficients. (When discussing BSBS, *p*_44_ should also be included). In diamond, (*p*_11_ *, p*_12_) = (−0.277, 0.058), whereas in silicon, (*p*_11_ *, p*_12_) = (0.09, −0.017)^7^. Thus, the *p*_11_ (or *p*_12_) in diamond and silicon show opposite signs, which leads to the opposite direction of ES forces.

**Figure 4:**
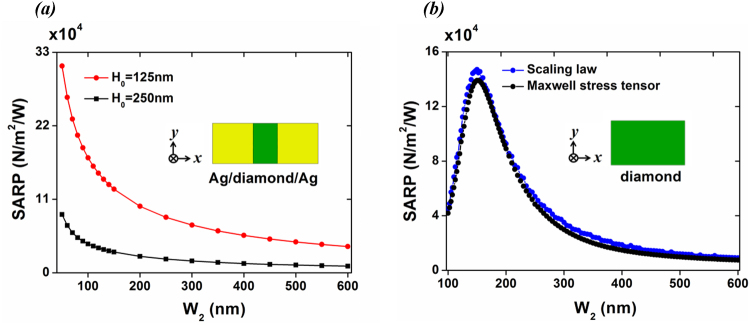
(**a**) SARP of GSP waveguide calculated by Maxwell stress tensor as the width of the dielectric gap *W*_2_ is varied; strong optical field gradients existed in the dielectric gap. (**b**) SARP of diamond-only waveguide calculated by Maxwell stress tensor (black dots) and the scaling law^24^ (blue dots). Here the height of the waveguide is fixed as *H*_0_ = 250 nm. Note that the polarization of the considered photonic mode in this case is TE-like, as discussed in ref.^7^. The incident wavelength is 637 nm, corresponding to the zero phonon line emission produced by nitrogen vacancy that can be embedded in diamond.

Further, we compare the SARP in hybrid metal/dielectric/metal and individual diamond-only waveguides to illustrate the force enhancement to the RP term. As depicted in Fig. 4(a), the SARP decreases exponentially with the increment of the gap width; specifically, for *H*_0_ = 125 nm, SARP is significantly enhanced (~10^5^ N/m^2^/W) as *W*_2_ becomes less than 150 nm. As noted, the phenomenon of observing enhanced SARP existing at boundaries of small dielectric gap is quite similar to the nano-scale optical trapping as reported in ref.^23^. This could be explained by the fact that the hybrid plasmonic waveguides allow sub-wavelength light confinement and significant optical field gradients. As a comparison, the SARP in diamond-only wire is shown in Fig. 4(b), in which the peak is on the order of ∼10^5^ N/m^2^/W. When the value of *W*_2_ goes below 100 nm, the SARP is much less than 4 × 10^4^ N/m^2^/W due to the poor confinement of photonic modes since diffraction limit is reached; if the value of *W*_2_ goes further beyond 180 nm, due to the limited modal expansion, the SARPs become much weaker^22^. Here, we have verified the SARP obtained from Maxwell stress tensor in dielectric material, using calculations based on scaling law: ${\overline{f}}^{rp}=({n}_{g}-{n}_{p})/(c\cdot {A}_{wg})$ ^24^. Note that *n*_*g*_ = *c/v*_*g*_, *n*_*p*_ = *c/v*_*p*_, where *c* is the speed of light in vacuum, and *v*_*g*_ (*v*_*p*_) is the group (phase) velocity. As seen in Fig. 4(b), the two curves agree well; the small deviation observed is caused by the limited resolution in differentiating angular frequency of optical modes. Since the scaling law is only valid for loss-less dielectric material, it is no longer feasible to calculate the radiation pressures in GSP waveguides which produce metallic losses. In the following, we apply the Maxwell stress tensor to treat the FSBS gain contribution from RP.

For a specific elastic mode with wavevector **K** (in FSBS, **K **≈ 0), the peak SBS gain can be given as^8^:

6 $$G=\omega \cdot Q\cdot {|\langle {\bf{f}},{\bf{u}}\rangle |}^{2}/2{k}_{eff}$$

7 $$\langle {\bf{f}},{\bf{u}}\rangle =\sum _{{\rm{m}}}\sum _{{\rm{n}}}\langle {{\bf{f}}}_{{\bf{n}}},{{\bf{u}}}_{{\bf{m}}}\rangle =\sum _{{\rm{m}}}\sum _{{\rm{n}}}\int {{{\bf{f}}}_{{\bf{n}}}}^{\ast }\cdot {{\bf{u}}}_{{\bf{m}}}\,d{A}_{wg}$$

where **u** denotes the mechanical displacement, **m**, **n** = *x or y*, *ω* is the angular frequency of optical mode, *k*_*eff*_ is the stiffness per unit length and **f** is the optical force that is normalized by power. Note that the spatial overlap integral in Eqn. (7) should include all individual integrals that contributed from individual force **f**_**n**_. The individual spatial overlap has both amplitude term and phase term; the former decides the maximal contribution whereas the latter leads to destructive or constructive interference. Basically, **f** = **f** ^**es**^ + **f** ^**rp**^, and the overall gain is contributed from both ES and RP terms. In the following discussion, we will find that both forces interfere by adding constructively or destructively.

**Figure 5:**
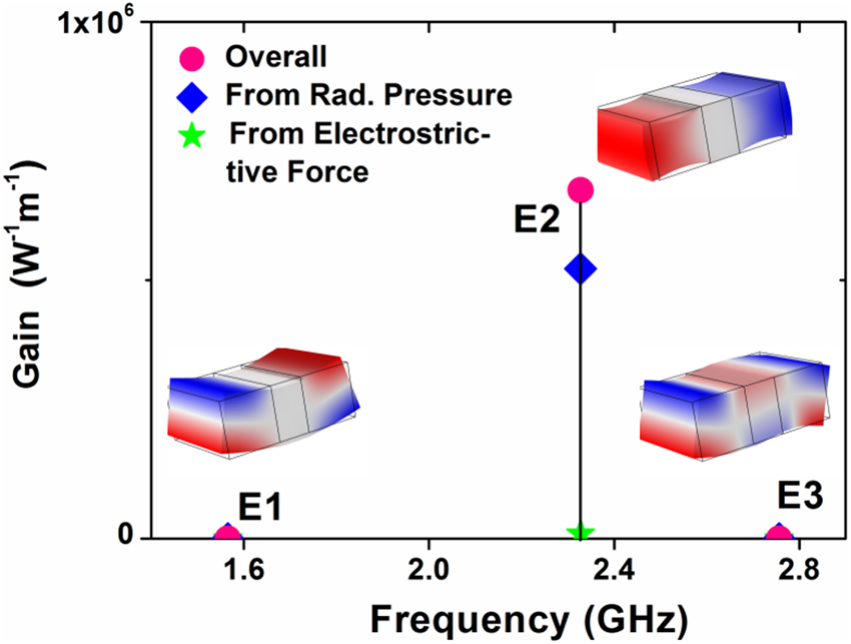
Brillouin spectrum of a GSP waveguide. The mode E2 produces significant overall gain, while the other two elastic eigenmodes show nearly zero effect. The incident wavelength is 637 nm, corresponding to the zero phonon line emission of nitrogen vacancy in diamond. The graph also presents gain contributions from ES forces (green star) and RP forces (blue diamond). Parameters are given as: *H*_0_ = 250 nm, *W*_2_ = 150 nm. The color of the modes means the sign of *x*-component of the displacement field: **u**_**x**_ (blue: negative, red: positive). Note that the periodic boundary conditions have been applied to two side walls which are in *x-y* plane, as shown in Fig. 1. Here the phonon wavevector **K** = 0.

Figure 5 shows three elastic modes and the corresponding FSBS gains. The modes E1 and E3 show odd symmetry to the plane *y* = *H*_0_/2; mode E2, also known as Lamé mode^25^, shows even symmetry; the Lamé mode E2 can be also considered as the surface acoustic wave which travels along the interface of metal and dielectric material as reported in ref.^26^.

Since the cross-sectional shape of the silver layer is assumed to be square, due to the odd symmetry to the plane *y* = *H*_0_/2, the **u**_**x**_ will be cancelled out along the vertical boundaries of diamond in cases of E1 and E3, but it will not be zero in Lamé mode E2. According to Eqns (6) and (7), only E2 can produce net FSBS gain. It can also be found that, RP (gain from RP ~5.2 × 10^5^ W^−1^ m^−1^) forces play a dominant role in contributing the overall FSBS gain which reaches 6.7 × 10^5^ W^−1^ m^−1^. In the case of diamond-only waveguide (see the cross-sectional view in Fig. 4(b)), the FSBS gain is only 2.8 × 10^3^ W^−1^ m^−1^. Also the gain contribution from RP is around ~2 × 10^2^ W^−1^ m^−1^, which is three orders smaller than that in GSP waveguide. The geometrical parameters in individual diamond waveguide are assumed to be the same: (*H*_0_, *W*_2_) = (250, 150) nm.

**Figure 6:**
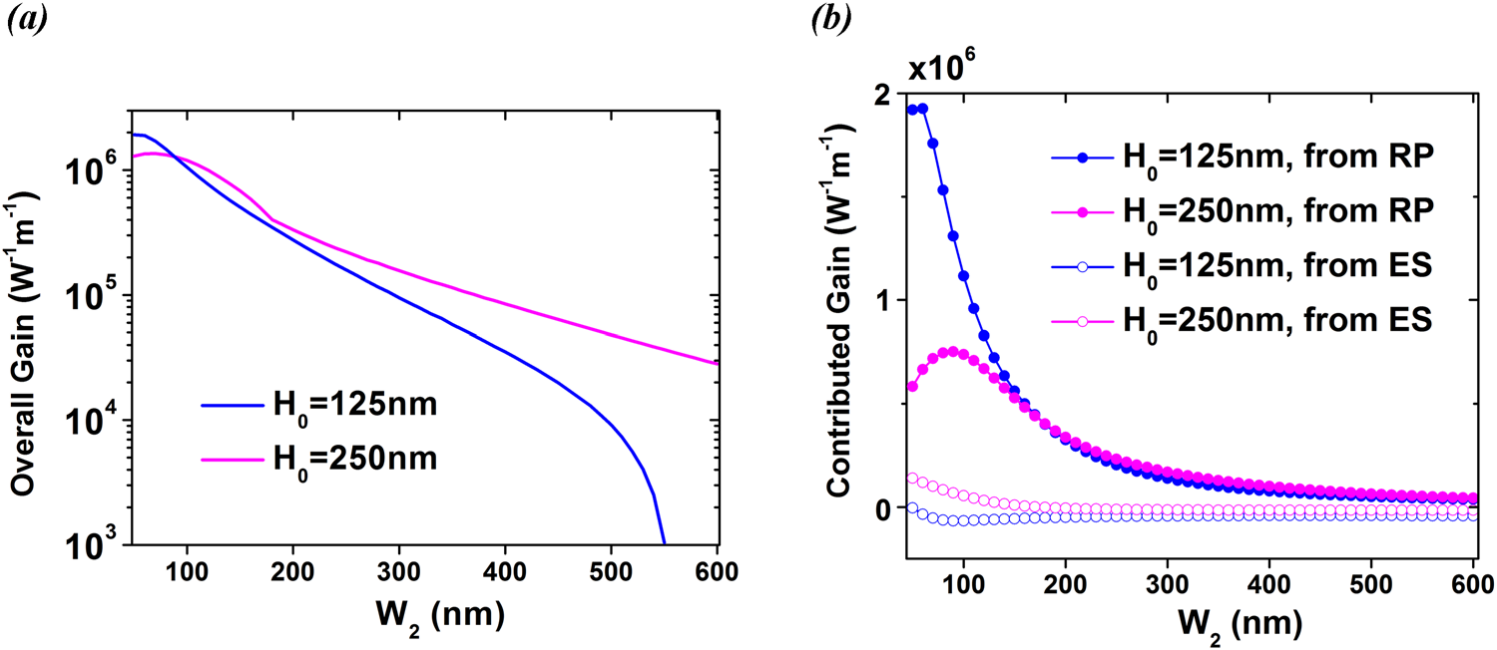
(**a**) Overall gains vs. the gap width *W*_2_ for the most promising mode E2 in the FSBS process. (**b**) Corresponding contributed gains from ES and RP forces vs. *W*_2_.

Further simulations have also been performed in order to examine the scale dependence of FSBS; we examine the FSBS gain versus the width of the diamond gap *W*_2_ when the height of the waveguide *H*_0_ is considered to be fixed. Figure 6(a,b) show the overall FSBS gains along with each contribution from RP and ES, when *W*_2_ is increased from 50 nm to 600 nm. To simplify calculations, the mechanical *Q* is assumed to be a constant of 1000, that is also assumed to be unaffected by the eigen-frequency change of mechanical modes, which is a typical assumption when discussing the SBS gain^7–9^. The simulated mechanical modes cover the frequency range from 4.2~4.7 GHz (for *H*_0_ = 125 nm) and 2.2~2.3 GHz (for *H*_0_ = 250 nm). All data points in Fig. 6 denote the maximal values of the computed FSBS gain.

As found in Fig. 6(a), the overall gain of the most promising mode E2 increases rapidly in the narrow dielectric gap. For example, the maximal overall gain for the case *H*_0_ = 125 nm can be 2 × 10^6^ W^−1^ m^−1^. In Fig. 6(b), it is easy to find that the dominant gain is contributed by RP. As discussed in Fig. 3, due to the fact that silver is a perfect conductor, the electric field at the boundary closer to the silver side is zero, which results in abrupt change of Maxwell stress tensor ${\overline{T}}_{xx}$. In other words, strong RP-induced nonlinearity could be produced in the GSP waveguides, regardless of the intrinsic photo-elastic property in the nonlinear material. As also noted in Fig. 6(b), the gain contribution from RP in the case of *H*_0_ = 125 nm is relatively larger than that of *H*_0_ = 250 nm in the regime *W*_2_ < 180 nm. The increased spatially averaged RP force in the waveguide with lower height shown in Fig. 4(a) leads to this result.

Figure 6(b) shows that the gain contribution from ES can be positive or negative. Several factors influence this result: (1) the *p*_11_ and *p*_22_ in diamond show opposite signs; (2) the main electric energy is carried by *E*_*x*_ component; and (3) for a given *W*_2_, the frequency of the elastic mode is decisively dependent of the cross-sectional area of silver: ${H}_{0}^{2}$. Due to the acoustic resistance mismatch between silver and diamond, elastic waves with varied eigen-frequencies will produce different energy losses at the investigated boundaries, which in turn affects the displacement field distributions **u**_**x**_, **u**_**y**_. The linear sum of the spatial overlaps in *x* and *y* directions as shown in Eqn. (7) will decide the sign of the ES gain.

The interference of RP (positive gain) and ES (positive or negative gain) will produce constructive or destructive overall gain. As an example, it can be found that the overall gain in case of *H*_0_ = 125 nm decreases rapidly to 10^3^ W^−1^ m^−1^ at *W*_2_ = 550 nm, due to the negative gain introduced by ES forces. Future work on promoting the overall FSBS gain thus could consider (1) selecting materials with positive and large *p*_11_ (e.g. silicon), or (2) decreasing the height/width of the gap in the waveguide in order to bring about a positive ES gain.

**Figure 7:**
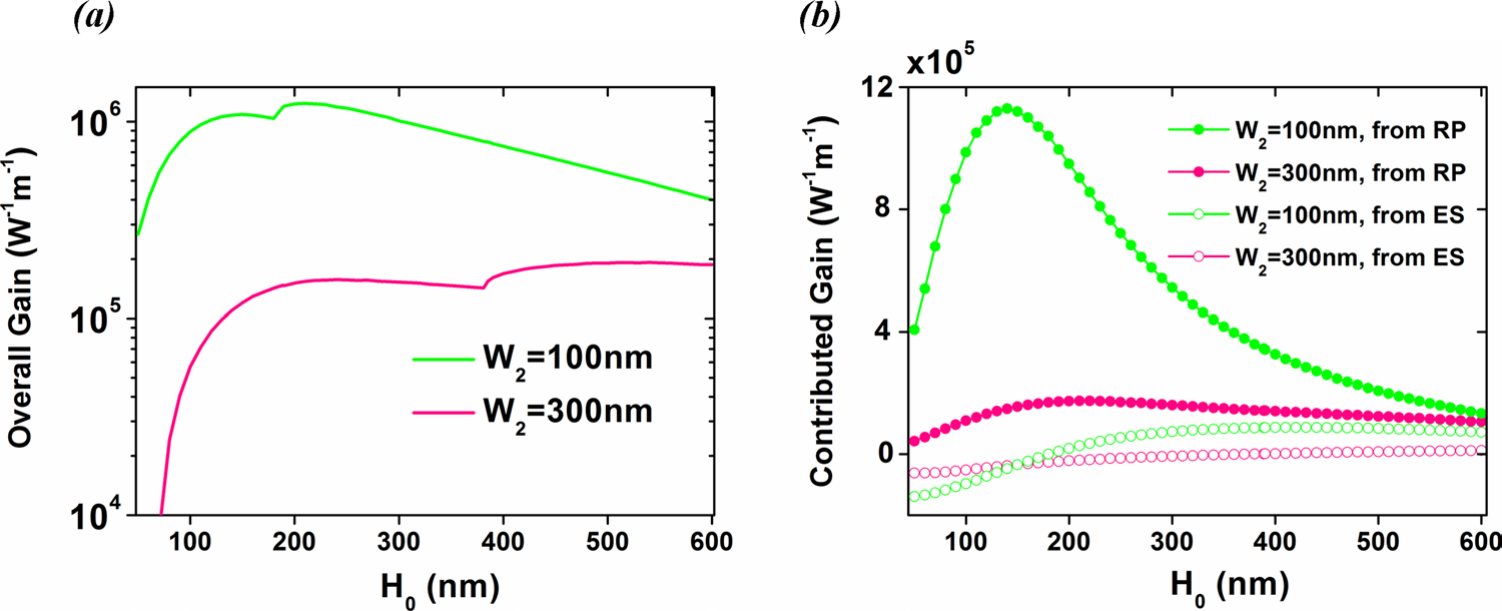
(**a**) Overall gains vs. the height of the dielectric material *H*_0_ for the most promising mode E2 in the FSBS process. (**b**) Corresponding contributed gains from ES and RP forces, as *H*_0_ varies.

The interference effect can also be observed in Fig. 7(a), which displays the overall Brillouin gains versus the height *H*_0_ of the dielectric material. Both curves show dips indicating that the ES contribution turns from negative to positive. For example, the overall gain in the case of *W*_2_ = 300 nm increases by one order of magnitude when *H*_0_ increases from 50 nm to 385 nm. Above (below) *H*_0_ = 385 nm, the ES contribution turns to be positive (negative) (see Fig. 7(b)) which results in constructive (destructive) interference. Also, larger gap width *W*_2_ provides flatter but lower gain contributions, hence lower maximal overall gain. As seen in Fig. 7(a), the overall gain could reach above 10^6^ W^−1^ m^−1^ for *W*_2_ = 100 nm and a wide range of *H*_0_: 110 nm < *H*_0_ < 300 nm.

A comparison between the proposed design and other reported designs is summarized in Table 1. It shows that the FEM is a powerful tool in simulating Brillouin scattering and predicting the overall Brillouin gain in various waveguides (e.g. line^7,9^, membrane^5^ and slot waveguides^8,27,28^). As reported in ref.^5^, the simulated Brillouin overall gain based on FEM agrees with the experimental result. Moreover, Brillouin gains in suspended structures^7–9,27^ are reported to be higher than that in quasi- or un-suspended structures^5,28,29^. The conventional designs are built upon high-index-contrast dielectric materials (e.g. silicon), which lead to relatively strong optical confinement in the line^5,7,9^, slot waveguides^8,27,28^ and resonators^30^. The optical index-contrast in diamond is low; Brillouin gain in suspended diamond-only waveguide as shown in this paper is calculated to be 2.8 × 10^3^ W^−1^ m^−1^, which is one order of magnitude smaller than that in suspended silicon-only waveguides as reported in ref.^7^ and ref.^9^. However, our design shows that by utilizing strong electric field enhancement that is induced by the sub-wavelength gap surface plasmon confinement, the overall Brillouin gain in our hybrid plasmonic waveguide design will surpass the gain in conventional designs^5,7–9,27–29^ by two to three orders of magnitude. It is noted that the Brillouin gain as reported in ref.^30^ for resonator is in the same order obtained for our proposed waveguide structure. However, the result in ref.^30^ is obtained through a microsphere, which employed an ultra-high-Q (4.9 × 10^8^) resonator, while it is known that a high quality factor resonator can achieve much higher gain than a waveguide; therefore, for waveguide applications, our proposed structure shows significantly better performance. The strong photon-phonon coupling in the proposed diamond GSP waveguide may help promote the study on spin dynamics of NV^−^ center that is driven by mechanical oscillators^31^.

**Table 1:** Comparison of the Brillouin gain between the proposed diamond plasmonic waveguide design and other designs. Note that *G*_*cal*_ (*G*_*exp*_) denotes the calculated (experimental) overall Brillouin Gain.

Structure	Material	Method	Overall Brillouin Gain *G* (W^−1^ m^−1^)
Plasmonic waveguide (this work)	diamond, silver	FEM	maximal ***G***_***cal***_ > 10^6^, for FSBS
Line waveguide^9^	silicon	FEM	***G***_***cal***_ = 1.72 × 10^4^, for FSBS
Line waveguide^7^	silicon	FEM	***G***_***cal***_ = 2.30 × 10^4^, for FSBS
Membrane waveguide^5^	silicon, silicon nitride	FEM and experimental method	***G***_***cal***_ = 2570 ± 540, for FSBS ***G***_***exp***_ = 2750 ± 1200, for FSBS
Slot waveguides^8^	silicon	FEM	maximal ***G***_***cal***_ = 4.5 × 10^4^, for FSBS
Slot waveguides^27^	silicon	FEM	***G***_***cal***_ = 5.0 × 10^4^, for FSBS
Hybrid slot waveguides^28^	Chalcogenide glass (As_2_S_3_ glass), silicon	FEM	***G***_***cal***_ = 3300, for BSBS
Nanowire racetrack resonator^29^	silicon	experimental method	***G***_***exp***_ = 2084, for FSBS
Microsphere resonator^30^	silica	experimental method	***G***_***exp***_ = 4 × 10^6^

Lastly, we point out that the metallic loss may become the main obstruction toward testing the aforementioned numerical results. High gains are expected in backward Brillouin scattering (BWBS) as well^9,32^. In BWBS, both longitudinal and transverse phonons are generated. We expect that our proposed sandwich waveguide design is also suitable for generating high gain BWBS. In addition, the phase matching condition may not necessarily be met when the thickness of silver film (e.g. nanolayer) gets closer to or becomes smaller than the wavelength of incident light^33^. Further studies on GSP waveguide to realize high FSBS gain may be useful to explore the interaction of surface plasmons and phonons.

## Conclusions

We have studied gap-surface-plasmon waveguides in search of strong optical forces in order to enhance the forward Brillouin scattering in diamond. In a narrow dielectric gap, we have numerically obtained efficient FSBS for a fundamental Lamé mode. The metallic loss may be an important limiting factor in testing our numerical results. The maximum SBS gain in the gap-surface plasmonic diamond waveguide could reach up to 10^6^ W^−1^ m^−1^, which is hard to achieve in standalone diamond-only photonic waveguides. It is also found that high gain is not decisively dependent on photoelastic coefficients of the dielectric material, since the radiation pressures at the boundaries of narrow gap play a dominant role in contributing to the overall gain. The large FSBS overall gain and feasibility of waveguide fabrication may make our design useful in applications involving surface plasmon enhanced stimulated Brillouin scattering, such as phonon laser, RF wave generation and optomechanical information processing in quantum system.

## Method

### Simulation method

Numerical simulations on full-vector analysis between photonic and elastic modes are performed by using finite-element method (COMSOL Multiphysics). Simulation results in Fig. 2, Fig. 3 and Fig. 4 are obtained by RF module included in COMSOL, and those in Fig. 5 are obtained by structural mechanics module included in COMSOL. Formula for calculating FSBS gain can be found in Eqn. (6). Results in Fig. 6 and Fig. 7 are obtained from Eqn. (6).
